# Management of edoxaban therapy and clinical outcomes in patients undergoing major or nonmajor surgery: a subanalysis of the EMIT-AF/VTE study

**DOI:** 10.1186/s12959-023-00568-2

**Published:** 2023-12-14

**Authors:** Christian von Heymann, Martin Unverdorben, Paolo Colonna, Amparo Santamaria, Manish Saxena, Thomas Vanassche, Sabine Köhler, Amanda P. Borrow, James Jin, Cathy Chen

**Affiliations:** 1 Department of Anaesthesia & Intensive Care Medicine, Emergency Medicine, and Pain Therapy, Vivantes Klinikum im Friedrichshain, Landsberger Allee 49, Berlin, 10249 Germany; 2 Daiichi Sankyo, Inc, Basking Ridge, NJ USA; 3 Polyclinic of Bari - Hospital, Department of Cardiology, Bari, Italy; 4 University Hospital Vinalopo Hematology Department, Alicante, Spain; 5 Barts NIHR Cardiovascular Biomedical Research Centre, London, UK; 6 Department of Cardiovascular Sciences, University Hospitals (UZ) Leuven, Leuven, Belgium; 7 Serenis GmbH, Munich, Germany

**Keywords:** Periprocedural management, Direct oral anticoagulation, Edoxaban interruption, Atrial fibrillation, EMIT-AF/VTE, Major Surgery, Nonmajor Surgery

## Abstract

**Background:**

Optimising periprocedural management of direct oral anticoagulation in patients with atrial fibrillation on chronic treatment undergoing major surgeries is an important aspect of balancing the risk of surgery-related bleeding with the risk of thromboembolic events, which may vary by surgery type.

**Methods:**

This subanalysis of the prospective EMIT-AF/VTE programme assessed periprocedural-edoxaban management, according to physicians’ decisions, and bleeding and thromboembolic event rates in patients who underwent major vs. nonmajor surgeries. Edoxaban interruption and clinical outcomes were compared between major vs. nonmajor surgeries and between renal function subgroups (creatinine clearance [CrCL] ≤ 50 mL/min vs. > 50 mL/min).

**Results:**

We included 276 major and 512 nonmajor surgeries. The median pre- and postprocedural duration of edoxaban interruption in major vs. nonmajor surgeries was 4 vs. 1 days, whereas median duration of interruption for those with preprocedural-only and postprocedural-only interruption was 2 vs. 1 days and 2 vs. 0 days, respectively (*P* < 0.0001). Rates of all bleeding and clinically relevant nonmajor bleeding were numerically higher in major vs. nonmajor surgeries. Event rates (number of events per 100 surgeries) were low overall (< 6 events per 100 surgeries), independent of renal function subgroups.

**Conclusion:**

In this subanalysis of the EMIT-AF/VTE programme, periprocedural-edoxaban interruption was significantly longer in patients undergoing major vs. nonmajor surgery. This clinician-driven approach was associated with low rates of bleeding and thromboembolic events following both major and nonmajor surgeries.

**Trial registration:**

NCT02950168, registered October 31, 2016; NCT02951039, registered November 1, 2016.

## Introduction

Annually, approximately 10% of patients receiving chronic anticoagulation therapy undergo diagnostic or therapeutic procedures that are associated with bleeding risks and require therapy interruption [1]. In particular, patients scheduled for major surgery have a high risk of bleeding. With the growing use of direct oral anticoagulants (DOACs), physicians must optimise periprocedural-DOAC management to balance the risk of bleeding with that of thromboembolic events. While clinical guidelines recommend that surgeries with high bleeding risks (e.g., major surgeries) should utilise temporary DOAC interruption, there are many less invasive procedures that carry a relatively low bleeding risk and do not necessitate interruption [1, 2]. However, real-world data on the safety and periprocedural management of DOAC therapy in the setting of major surgeries with a high risk of bleeding are limited.

Previous studies have reviewed and assessed the pharmacological properties [3] and periprocedural management of DOACs, including rivaroxaban, dabigatran, and apixaban [4]. In the prospective, noninterventional Dresden registry, patients who underwent major procedures were significantly more likely to experience bleeding and major cardiovascular (CV) events as well as CV death when compared with patients who underwent minimal and minor procedures [5]. Additionally, in the prospective, nonrandomised PAUSE trial, rates of major bleeding were higher in patients undergoing a high-bleeding-risk procedure treated with apixaban (2.96%) or rivaroxaban (2.95%) compared with dabigatran-treated patients (0.88%) [6]. Notably, a subgroup of dabigatran-treated patients with creatinine clearance (CrCL) < 50 mL/min undergoing a high-bleeding-risk procedure had slightly longer preprocedural DOAC interruption compared with patients treated with apixaban or rivaroxaban undergoing a high-bleeding-risk procedure [6].

Edoxaban is a DOAC approved for the prevention of stroke and systemic embolic events (SEEs) in patients with nonvalvular atrial fibrillation (AF) and for the prevention and treatment of venous thromboembolism (VTE) [7–10]. Real-world data regarding periprocedural-edoxaban management are limited, especially in patients undergoing major surgeries.

The EMIT-AF/VTE programme (Edoxaban Management in Diagnostic and Therapeutic Procedures) was designed to investigate bleeding and thromboembolic events prospectively in patients with AF or VTE treated with edoxaban and undergoing procedures of varying risk levels [11, 12]. Primary analysis of the EMIT-AF/VTE data showed low rates of periprocedural major bleeding, clinically relevant nonmajor bleeding (CRNMB), acute thromboembolic events, and acute coronary syndrome in edoxaban-treated patients who underwent a wide range of diagnostic and therapeutic procedures [13]. The objective of this subanalysis is to compare the periprocedural management of edoxaban and clinical outcomes in patients who underwent major vs. nonmajor surgeries.

## Methods

### Study design

The design and overall results of the Global EMIT-AF/VTE programme (NCT02950168, NCT02951039) are published [11, 13]. EMIT-AF/VTE is a multicentre, prospective, observational programme conducted in Europe and Asia in accordance with the Declaration of Helsinki and with local Institutional Review Board approvals. Written informed consent was obtained from all participants prior to enrolment. The periprocedural management of edoxaban therapy was at the discretion of the investigator, including any decision regarding treatment interruption and the timing/duration of any interruption.

### Patient recruitment

EMIT-AF/VTE programme enrolment commenced in December 2016 and completed in August 2020 for the countries reported here. Patients were recruited from Belgium, Germany, Italy, the Netherlands, Portugal, Spain, the UK, South Korea, and Taiwan. Eligible patients were ≥ 18 years of age, had AF or VTE, were treated with edoxaban according to the local labels, were not enrolled in any interventional study concurrently, and underwent any type of diagnostic or therapeutic procedure [11, 13]. Surgeries, and therefore patients, were excluded from the analysis if there were multiple surgeries on the same day, missing surgery dates, and/or surgery date was more than 14 days after last edoxaban dose.

## Observations and outcomes

Observations, including edoxaban interruption and clinical event data, were recorded from 5 days before the procedure until 30 days after the procedure. To enhance data capture, patients received a memory aid booklet at study enrolment, which was reviewed at the end of the study. Edoxaban therapy was considered as uninterrupted if treatment was administered on each day of the observation period, including at any time on the day of the procedure. Any interruption of edoxaban treatment was recorded as the number of days without administration of edoxaban (preprocedural days [5 days before procedure and at procedure day] and/or postprocedural days [within 30 days after procedure]).

The primary safety outcome was the incidence of major bleeding, as defined by the International Society of Thrombosis and Haemostasis (ISTH) [14, 15]. Secondary outcomes included evaluation of periprocedural-edoxaban interruption, incidence of acute coronary syndrome (ACS), CRNMB, minor bleeding, all bleeding, all-cause death, CV death, and acute thromboembolic events (stroke, transient ischaemic attack, SEE). All major bleeding, CRNMB, ACS, and acute thromboembolic events were reviewed and unanimously adjudicated by the Steering Committee. Within each group, periprocedural-edoxaban interruption and clinical events were also analysed by renal function category (CrCL ≤ 50 vs. > 50 mL/min). The following parameters were documented at baseline: concomitant medications; HAS-BLED (Hypertension, Abnormal renal/liver function, Stroke, Bleeding history or predisposition, Labile international normalised ratio, Elderly, Drugs/alcohol concomitantly) score; CHA_2_DS_2_-VASc (Congestive heart failure, Hypertension, Age ≥ 75 [doubled], Diabetes, Stroke [doubled], Vascular disease, Age 65–74 years, and Sex category [female]) score; renal function; details of edoxaban treatment; diagnostic/therapeutic procedures; and medical history.

### Classification of surgeries

Major surgeries were classified by a combination of criteria used in the Dresden registry and PAUSE study: relevant tissue trauma and high bleeding risk; utilisation of general or neuraxial anaesthesia; major intracranial, neuraxial, thoracic, cardiac, vascular, abdominopelvic, or orthopaedic surgery; or other major cancer or reconstructive surgery [5, 16]. All major surgeries were considered high risk based on European Heart Rhythm Association (EHRA) bleeding risk levels, and nonmajor surgeries were assigned risk levels per EHRA periprocedural bleeding risk criteria.

### Statistical analysis

Baseline data are presented as frequencies and/or as summary statistics. *P*-values for baseline characteristics were calculated using Fisher’s exact test. Pre- and postprocedural edoxaban interruption and clinical outcomes were compared between major vs. nonmajor surgery groups and between renal function subgroups (CrCL ≤ 50 mL/min vs. CrCL > 50 mL/min, calculated using the Cockcroft-Gault equation); data are presented as summary statistics (n, mean, standard deviation [SD]) for numerical parameters and absolute and relative frequencies for duration of interruption between major vs. nonmajor surgeries. Clinical event rates are presented as number of events per 100 surgeries. Clinical outcomes analyses were descriptive and exploratory; no statistical comparisons were made between subgroups. Edoxaban interruption duration data included all patients, both with and without interruption, to avoid selection bias. *P*-values for duration of edoxaban interruption were calculated using the Wilcoxon test.

## Results

### Patient demographics and baseline characteristics

**Fig. 1:**
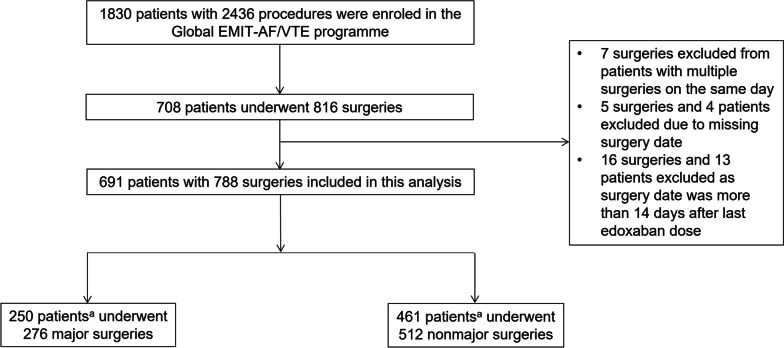
Patient disposition flow chart.  ^a^20 patients had both major and nonmajor surgeries and were counted in both groups

**Fig. 2:**
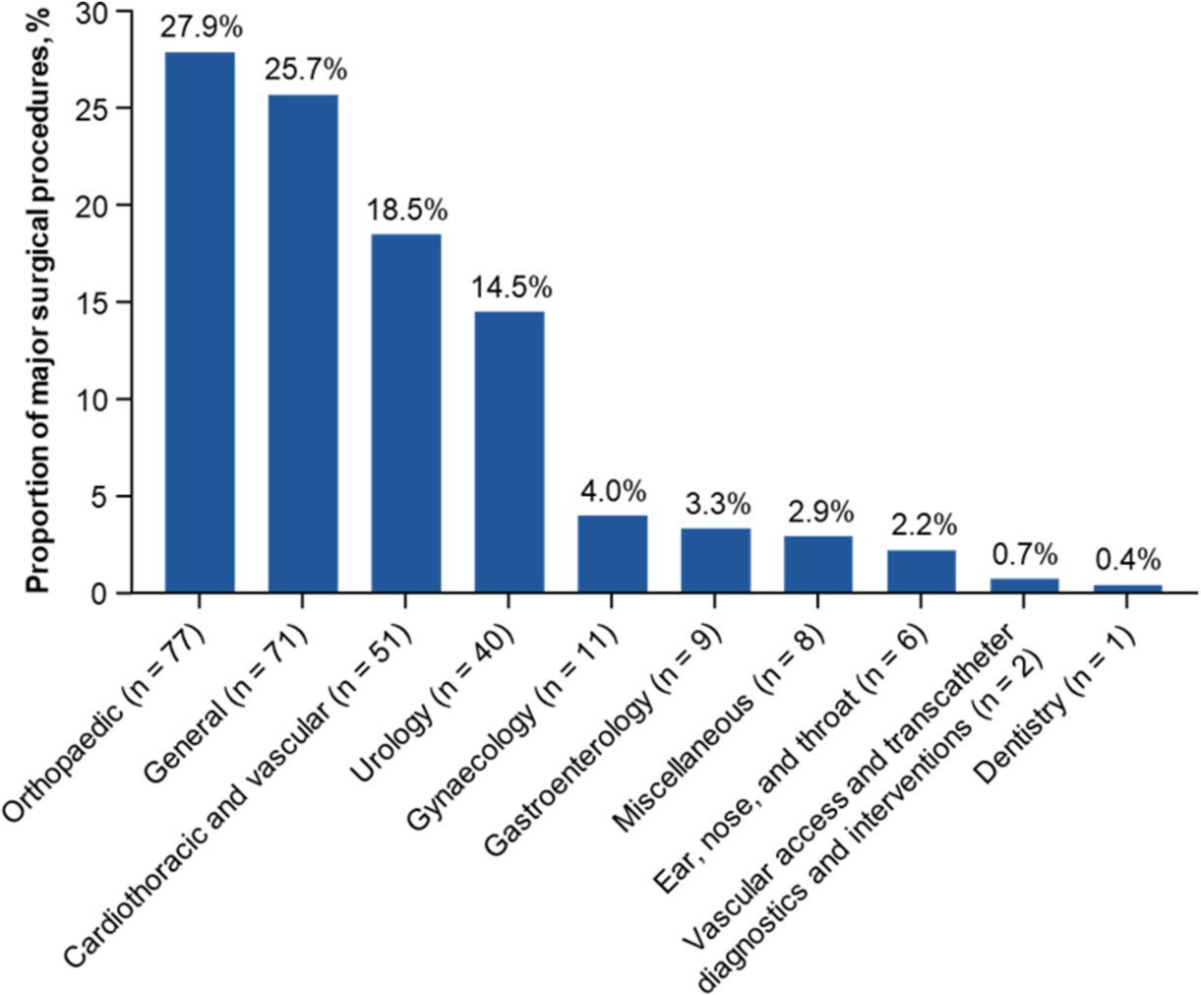
Proportion of major surgeries (*n* = 276) by surgery type. N, number of surgeries

Overall, 1830 patients who underwent 2436 procedures were enrolled in the Global EMIT-AF/VTE programme, of which 250 (35.2%) patients underwent major surgeries and 461 (64.8%) patients underwent nonmajor surgeries; 788 surgeries were analysed, as some patients underwent more than one surgery (Fig. 1). A total of 276 major surgeries were performed, with the most common being orthopaedic (27.9%), general (25.7%), or cardiothoracic/vascular (18.5%; Fig. 2). Patients who underwent major surgeries were significantly younger at enrolment (mean ± SD, 73.1 ± 8.8 years) than patients who underwent nonmajor surgeries (mean ± SD, 74.8 ± 9.7 years; *P* = 0.02; Table 1). A higher percentage of patients who underwent major surgeries were in the 65 to < 75 age group compared with those who underwent nonmajor surgeries. Patients who underwent major vs. nonmajor surgeries had similar baseline CHA_2_DS_2_-VASc (mean ± SD, 3.5 ± 1.5 vs. 3.6 ± 1.5) and HAS-BLED scores (mean ± SD, 2.0 ± 1.0 vs. 1.8 ± 1.1). The percentage of patients with impaired renal function (CrCL ≤ 50 mL/min) at baseline was 20.8% in the major surgery group and 24.9% in the nonmajor surgery group. Baseline CrCL was numerically higher for patients who underwent major surgeries (mean ± SD, 70.7 ± 25.9 mL/min) when compared with those who underwent nonmajor surgeries (mean ± SD, 66.3 ± 27.8 mL/min; *P* = 0.052). The percentages of patients undergoing major surgeries receiving 60 and 30 mg edoxaban were similar to the percentages of patients undergoing nonmajor surgeries (64.8% and 34.4% vs. 65.5% and 34.3%, respectively; Table 1).

**Table 1:** Baseline patient demographics and clinical characteristics

	Major surgery patients^a^ (*n* = 250)	Nonmajor surgery patients^a^ (*n* = 461)	*P*-value
**Age**^b^, years, mean ± SD	73.1 ± 8.8	74.8 ± 9.7	0.023
<65	37 (14.8)	55 (11.9)	
65 to < 75	103 (41.2)	146 (31.7)	
≥75	110 (44.0)	260 (56.4)	
**Sex**			
** Male**	153 (61.2)	276 (59.9)	0.729
** Female**	97 (38.8)	185 (40.1)	
**Weight**, kg, mean ± SD	75.6 ± 16.4	75.9 ± 16.2	0.808
**BMI**, kg/m^2^, mean ± SD	26.7 ± 4.4	27.0 ± 5.0	0.415
**Atrial fibrillation**	229 (91.6)	437 (94.8)	0.095
**CrCL**, mL/min, mean ± SD	70.7 ± 25.9	66.3 ± 27.8	0.052
≤50	52 (20.8)	115 (24.9)	
**Hypertension**	186 (74.4)	350 (75.9)	0.653
**Diabetes mellitus**	49 (19.6)	132 (28.6)	0.008
**Dyslipidaemia**	108 (43.2)	182 (39.5)	0.335
**Coronary heart disease**	41 (16.4)	86 (18.7)	0.454
**Valvular heart disease**	46 (18.4)	81 (17.6)	0.783
**Congestive heart failure**	33 (13.2)	64 (13.9)	0.800
**HAS-BLED score**, mean ± SD	2.0 ± 1.0	1.8 ± 1.1	0.134
**CHA**_**2**_**DS**_**2**_**-VASc score**, mean ± SD	3.5 ± 1.5	3.6 ± 1.5	0.476
**Edoxaban dose**			
30 mg/day	86 (34.4)	158 (34.3)	0.973
60 mg/day	162 (64.8)	302 (65.5)	
Other	2 (0.8)	1 (0.2)	
**Antiplatelet agents**	38 (15.2)	52 (11.3)	0.133

Data are shown as n (%) unless otherwise noted. *P*-values were calculated using Fisher’s exact test

*BMI* Body mass index, *CHA*_2_*DS*_2_*-VASc* Congestive heart failure, Hypertension, Age ≥ 75 (doubled), Diabetes, Stroke (doubled), Vascular disease, Age 65–74 years, and Sex category, CrCL Creatinine clearance, *HAS-BLED* Hypertension, Abnormal renal/liver function, Stroke, Bleeding history or predisposition, Labile international normalised ratio, Elderly, Drugs/alcohol concomitantly, *n* Number of patients, *SD* Standard deviation

^a^Baseline data for patients who underwent both major and nonmajor surgery on the same day were counted once in the major surgery group

^b^Age at the time of enrolment

### Periprocedural-edoxaban interruption

Periprocedural-edoxaban interruption was assessed in 276 major and 512 nonmajor surgeries. The number of major vs. nonmajor surgeries with pre- and postprocedural interruption was 160 (58.4%) vs. 114 (22.3%), respectively (*P* < 0.0001; Table 2). The number of major vs. nonmajor surgeries with preprocedural interruption only was 47 (17.2%) vs. 222 (43.4%), respectively; 13 (4.7%) major surgeries and 16 (3.1%) nonmajor surgeries had postprocedural interruption only (Table 2). Of the major surgeries with edoxaban interruption, 37 (13.5%) had interruption on only day 0 (surgery day), while 26 (9.5%) had interruption on only days 0 and 1; no surgeries had interruption on only day 1.

**Table 2:** Edoxaban interruption by EHRA bleeding risk level and time of interruption relative to surgery

	Pre- and postprocedure	Preprocedure only	Postprocedure only	No interruption	Total
**Major surgery**^**a, b**^	160 (58.4)	47 (17.2)	13 (4.7)	54 (19.7)	274 (100)
**Nonmajor surgery**^**c**^	114 (22.3)	222 (43.4)	16 (3.1)	160 (31.3)	512 (100)
High risk	6 (46.2)	4 (30.8)	0	3 (23.1)	13 (100)
Low risk	38 (20.3)	70 (37.4)	6 (3.2)	73 (39.0)	187 (100)
Minor risk	69 (22.2)	148 (47.6)	10 (3.2)	84 (27.0)	311 (100)
Unknown	1 (100)	0	0	0	1 (100)

Data are shown as n (%)^d^

High-risk nonmajor surgeries include orthopaedic (hand surgery; *n* = 4), gastroenterology (tumour excision; *n* = 5); cardiothoracic and vascular (thoracotomy/thoracocentesis ± chest tubes; *n* = 3), and general surgery (solid organ resection; *n* = 1)

*EHRA* European Heart Rhythm Association, *n* Number of surgeries

^a^Median edoxaban interruption time for major surgeries was 4.0 days

^b^For 2 surgeries, data for edoxaban use on the date of the surgery are unavailable

^c^Median edoxaban interruption time for nonmajor surgeries was 1.0 day

^d^Percentages are relative to the total number of surgeries in each surgery group

**Fig. 3:**
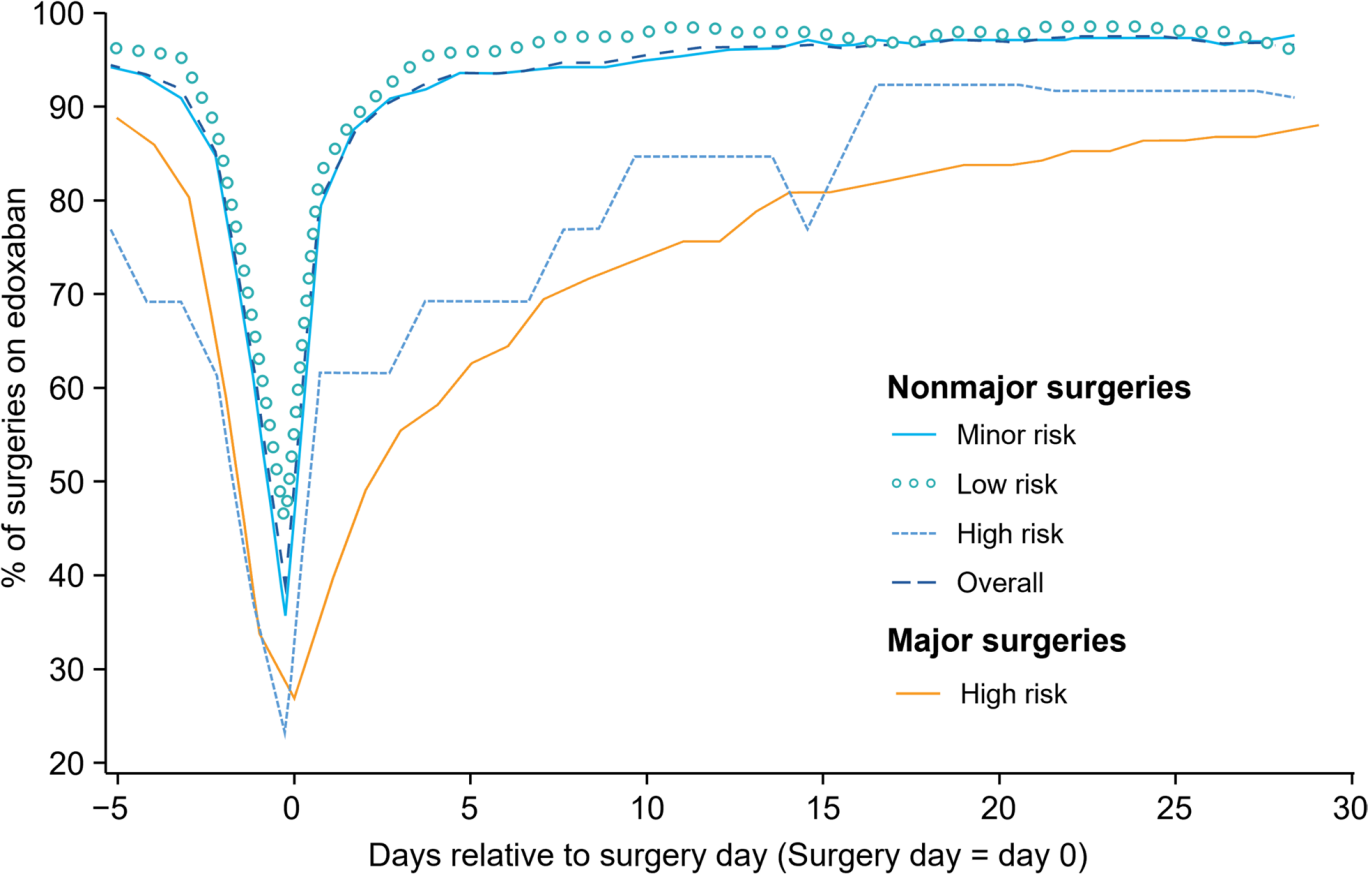
Periprocedural-edoxaban interruption over time.  Major surgeries were considered high risk based on EHRA bleeding risk levels. Nonmajor surgeries were assigned risk levels per EHRA procedural bleeding risk criteria.  EHRA, European Heart Rhythm Association

When including surgeries without interruption, the median duration of edoxaban interruption in major vs. nonmajor surgeries with pre- and postprocedural, preprocedural, or postprocedural interruption was 4 vs. 1 days, 2 vs. 1 days, or 2 vs. 0 days, respectively (*P* < 0.0001 for all; Table 3). Table 4 summarises preprocedural edoxaban interruption, excluding surgeries without interruption. The number of major and nonmajor surgeries without any interruption were 54 (19.7%) and 160 (31.3%), respectively. A protracted period of time before edoxaban resumption followed major and nonmajor surgeries with high bleeding risk (Fig. 3).

**Table 3:** Duration of edoxaban interruption (days) for major and nonmajor surgeries

Time of interruption relative to procedure^a^	Major surgeries (*n* = 274)^b, c^	Nonmajor surgeries^d^ (*n* = 512)	*P-*value from Wilcoxon test
**Pre- and postprocedure**			**< 0.0001**
Median	4	1	
Mean ± SD	9.0 ± 10.6	2.8 ± 5.3	
**Preprocedure**			**< 0.0001**
Median	2	1	
Mean ± SD	2.2 ± 1.8	1.3 ± 1.5	
**Postprocedure**			**< 0.0001**
Median	2	0	
Mean ± SD	7.0 ± 9.7	1.5 ± 4.7	

Data shown are number of days

*n* Number of surgeries, *SD* Standard deviation

^a^Groups include surgeries without interruption

^b^Postprocedural interruption, *n* = 267

^c^For 2 surgeries, data for edoxaban use on the date of the surgery are unavailable

^d^Postprocedural interruption, *n* = 507

**Table 4:** Preprocedural edoxaban interruption for major and nonmajor surgeries, excluding procedures without interruption

	Major surgeries		Nonmajor surgeries	
Time of interruption relative to surgeries	Number of surgeries	Duration of preprocedural interruption, median^a^	Number of surgeries	Duration of preprocedural interruption, median^a^
**Pre- and postprocedure interruption**	160	3	114	2
**Preprocedure interruption only**	47	2	222	1
**Postprocedure interruption only**	13	NA	16	NA

The number of major and nonmajor surgeries that had no edoxaban interruption were 54 and 160 surgeries, respectively

*NA* Not applicable

^a^This table summarises data collected over the preprocedural window (days − 5 to 0); therefore, duration of interruption was not calculated for the postprocedure interruption

### Clinical outcomes

Major bleeding rates (number of events per 100 surgeries) were similar for major vs. nonmajor surgeries (0.4 vs. 0.6, respectively; Table 5). Rates of all bleeding in major vs. nonmajor surgeries were 4.3 vs. 3.3, and rates of CRNMB were 1.4 vs. 0.2 in major and nonmajor surgeries, respectively (Table 5). Overall, 2 thromboembolic events (1 stroke in nonmajor surgery group and 1 SEE in major surgery group) and 2 deaths (1 sepsis and 1 malignancy in major surgery group) were reported.

**Table 5:** Clinical outcomes for major or nonmajor surgeries

	Major surgeries (*n* = 276)	Nonmajor surgeries (*n* = 512)
**All bleeding**	12 (4.3)	17 (3.3)
**Major bleeding**^a^	1 (0.4)	3 (0.6)
**CRNMB**	4 (1.4)	1 (0.2)
**Minor bleeding**	7 (2.5)	13 (2.5)
**Acute coronary syndrome**	0	0
**Stroke**	0	1 (0.2)
**Transient ischaemic attack**	0	0
**Systemic embolic events**	1 (0.4)	0
**All-cause death**	2 (0.7)	0
**CV death**	0	0

Data are shown as n (number of events per 100 surgeries)

*CRNMB* Clinically relevant nonmajor bleeding, *CV* Cardiovascular, *n* Number of surgeries

^a^International Society of Thrombosis and Haemostasis definition

### Periprocedural-edoxaban interruption and clinical outcomes stratified by renal function

Patients with CrCL ≤ 50 mL/min and with CrCL > 50 mL/min had similar rates of pre- and postprocedural interruption, preprocedural-only interruption, and postprocedural-only edoxaban interruption (Table 6). Of the major surgeries with edoxaban interruption, treatment resumption was slower in patients with CrCL ≤ 50 mL/min when compared with patients with CrCL > 50 mL/min. In major surgeries with preprocedural interruption, the number of patients with edoxaban resumption ≥ 5 days after the surgery day was 28 (59.6%) in the CrCL ≤ 50 mL/min group and 67 (47.5%) in the CrCL > 50 mL/min group. Overall, the timing of edoxaban resumption did not differ by renal function category.

**Table 6:** Major or nonmajor surgeries edoxaban interruption by renal function

	Pre- and postprocedure	Preprocedure only	Postprocedure only	No interruption	Total
**Major surgery**^a,b^	160 (58.4)	47 (17.2)	13 (4.7)	54 (19.7)	274 (100)
CrCL ≤ 50	35 (54.7)	12 (18.8)	4 (6.3)	13 (20.3)	64 (100)
CrCL > 50	108 (57.4)	33 (17.6)	9 (4.8)	38 (20.2)	188 (100)
CrCL unknown	17 (77.3)	2 (9.1)	0	3 (13.6)	22 (100)
**Nonmajor surgery**^c^	114 (22.3)	222 (43.4)	16 (3.1)	160 (31.3)	512 (100)
CrCL ≤ 50	26 (19.4)	54 (40.3)	3 (2.2)	51 (38.1)	134 (100)
CrCL > 50	71 (23.5)	137 (45.4)	9 (3.0)	85 (28.1)	302 (100)
CrCL unknown	17 (22.4)	31 (40.8)	4 (5.3)	24 (31.6)	76 (100)

Data are shown as n (%)^d^

*CrCL *Creatinine clearance (mL/min), *n* Number of surgeries

^a^Median edoxaban interruption time for major surgeries, including surgeries without interruption, was 4.0 days

^b^For 2 surgeries, data for edoxaban use on the date of the surgery are unavailable

^c^Median edoxaban interruption time for nonmajor surgeries, including surgeries without interruption, was 1.0 days

^d^Percentages are relative to the total number of surgeries in renal function subgroups

Of patients who underwent major surgeries, the rate of all bleeding events was numerically lower in patients with CrCL ≤ 50 mL/min than in patients with CrCL > 50 mL/min (1.6 vs. 5.3); both deaths reported in the study occurred in patients with CrCL > 50 mL/min (Table 7). Of patients who underwent nonmajor surgeries, the rate of all bleeding events was numerically higher in patients with CrCL ≤ 50 mL/min than in patients with CrCL > 50 mL/min (5.2 vs. 2.0).

**Table 7:** Clinical outcomes for major or nonmajor surgeries by renal function

	Major surgeries			Nonmajor surgeries		
	CrCL ≤ 50 (*n* = 64)	CrCL > 50 (*n* = 189)	CrCL unknown (*n* = 23)	CrCL ≤ 50 (*n* = 134)	CrCL > 50 (*n* = 302)	CrCL unknown (*n* = 76)
**All bleeding**	1 (1.6)	10 (5.3)	1 (4.3)	7 (5.2)	6 (2.0)	4 (5.3)
**Major bleeding**^a^	0	1 (0.5)	0	1 (0.7)	1 (0.3)	1 (1.3)
**CRNMB**	0	4 (2.1)	0	1 (0.7)	0	0
**Minor bleeding**	1 (1.6)	5 (2.6)	1 (4.3)	5 (3.7)	5 (1.7)	3 (3.9)
**ACS**	0	0	0	0	0	0
**Stroke**	0	0	0	1 (0.7)	0	0
**TIA**	0	0	0	0	0	0
**SEE**	0	1 (0.5)	0	0	0	0
**All-cause death**	0	2 (1.1)	0	0	0	0
**CV death**	0	0	0	0	0	0

Data are shown as n (number of events per 100 surgeries)

*ACS* Acute coronary syndrome, *CrCL* Creatinine clearance (mL/min), *CRNMB* Clinically relevant nonmajor bleeding, *CV* Cardiovascular, *n* Number of surgeries, *SEE* Systemic embolism event, *TIA* Transient ischaemic attack

^a^International Society of Thrombosis and Haemostasis definition

## Discussion

This subanalysis of the Global EMIT-AF/VTE programme assessed the periprocedural management of edoxaban and the occurrence of bleeding and thromboembolic events in edoxaban-treated patients who underwent major or nonmajor surgeries. To the authors’ knowledge, this analysis is the first to report treatment interruption and clinical events in patients treated with edoxaban undergoing major or nonmajor surgeries. Baseline CHA_2_DS_2_-VASc score, HAS-BLED score, and CrCL were similar between patients who underwent major and nonmajor surgeries. While major surgeries had a longer period of edoxaban interruption compared to nonmajor surgeries, low rates of all bleeding, major bleeding, CRNMB, and thromboembolic events were observed in both groups. These results suggest that longer periprocedural edoxaban interruption for patients undergoing major surgeries may help mitigate the bleeding and thromboembolic risk in this group.

The periprocedural management of DOAC treatment focuses on reducing the risk of bleeding without increasing the risk of thromboembolic events. Conversely, prolonged interruption of DOAC therapy may increase the risk of thromboembolism, most importantly ischaemic stroke, whereas an interruption that is too brief may increase the risk of bleeding. In our study, edoxaban therapy was not interrupted for 54 (19.6%) of the major surgeries, which may have been due in part to clinician interpretation of minor hemorrhagic risk and in part to the fact that 3 of these were unplanned (emergency/urgent) surgeries. Major surgeries carry a higher risk of bleeding, and most recommendations suggest longer interruption times compared with low- or minor-bleeding-risk surgeries [17]. In line with these recommendations, the current study of routine clinical practice found major surgeries had longer edoxaban interruption times when compared with nonmajor surgeries. Notably, there were only 24.5% of major surgeries without any preprocedural interruption; 23.0% of major surgeries had one day or less of postprocedural interruption (interruption on day 0 and/or day 1). This agrees with findings from a subanalysis of the prospective Dresden registry, which reported data on 863 surgical or interventional procedures in DOAC-treated patients receiving predominantly rivaroxaban [5]. Of the procedures reported, 87 (10.1%) were major surgical procedures, 641 (74.3%) were minor procedures, and 135 (15.6%) were minimal procedures [5]. Despite having a smaller percentage of major procedures compared with our study, the Dresden study was similar to our analysis in that DOAC use was not interrupted in 22% of patients undergoing surgeries, and the majority of procedures were performed with DOAC interruption [5].

For patients participating in the Dresden registry, bleeding and cardiovascular event rates were low, similar to this subanalysis [5]. Notably, the present study stratified patients undergoing major and nonmajor surgeries by pre- and postprocedural interruption, whereas the Dresden study analyzed periprocedural-DOAC interruption in major, minor, and minimal procedures [5]. The Dresden study also did not stratify patients by time of interruption relative to the procedure, nor did it specify whether DOAC use on the day of a procedure was considered uninterrupted [5]. Overall, bleeding and cardiovascular events were < 6% for all procedures; patients who underwent minimal and minor procedures vs. those who underwent major procedures had significantly higher rates of any bleeding (2.2% and 4.5% vs. 16.1%), major bleeding (0% and 0.5% vs. 8.0%), CRNMB (1.5% and 3.1% vs. 8.0%), major CV events (0% and 0.8% vs. 4.6%), or CV death (0% and 0.2% vs. 2.3%) at day 30 ± 5 after the procedure [5].

Additionally, results from this study are comparable with those from the PAUSE study. However, while the PAUSE study used a predefined interruption protocol for surgeries with different bleeding risks, the EMIT-AF/VTE study left the periprocedural-edoxaban management to the attending clinician without any influence of a study protocol [11, 16]. Therefore, the results of this analysis with low bleeding and ischaemic events in major surgeries suggest that clinicians made the right decision to confine the risk of bleeding in high-risk major surgeries while not increasing the risk of preoperative ischaemic events. Furthermore, compared with the PAUSE trial, this study used a stronger definition for major surgeries that combined the criteria utilised in both the PAUSE and Dresden studies; this improved definition may reduce the risk of selection bias within our study [5, 11, 16].

In patients with AF, renal dysfunction is a risk factor for both thromboembolic and bleeding events [18, 19]. Current guidelines recommend a reduced dose of DOACs in patients with renal impairment (CrCL ≤ 50 mL/min) [20]. In the current study, treatment resumption was protracted in patients with CrCL ≤ 50 mL/min vs. CrCL > 50 mL/min; approximately 70% of patients with CrCL ≤ 50 mL/min resumed edoxaban by day 30 vs. 90% of patients with CrCL > 50 mL/min. With regards to clinical event rates, in a subanalysis of the ENGAGE AF-TIMI 48 trial, patients on a high-dose edoxaban regimen with moderately reduced renal function (CrCL 30–50 mL/min) had numerically lower rates of major bleeding when compared with patients with CrCL > 50 mL/min [21]. In the current study, the rate of all bleeding events for patients with CrCL ≤ 50 mL/min vs. those with CrCL > 50 mL/min undergoing major surgeries was numerically lower, while the rate of all bleeding events for patients with CrCL ≤ 50 mL/min vs. those with CrCL > 50 mL/min undergoing nonmajor surgeries was numerically higher. This may be due, in part, to a longer periprocedural interruption time in patients with CrCL ≤ 50 mL/min vs. those with CrCL > 50 mL/min undergoing major surgeries, whereas in the nonmajor surgery group, there was no difference in interruption duration between renal function subgroups. These results support the safety of the clinician-driven, edoxaban-management regimen in vulnerable populations, such as patients with renal impairment. However, bleeding event rates (number of events per 100 surgeries) were low overall, regardless of renal function or surgery group (< 6 for all outcomes).

Limitations of this subanalysis include the lack of a DOAC-comparator arm and the lack of formal statistical comparisons between groups for the periprocedural management of edoxaban and clinical outcomes. Additionally, edoxaban management was not standardised, as it was at the discretion of the investigator; however, this enabled patient-individualised treatment. EMIT-AF/VTE is a global programme with data from 326 centres comprising a large number of patients undergoing a wide range of major or nonmajor surgeries in routine clinical practice, including a high percentage of patients (20.8%) with CrCL ≤ 50 mL/min, representing a strength of this analysis. As a large observational study, these data complement randomised controlled trial data, reflecting edoxaban management in current clinical practice without the guidance of a predefined study protocol.

## Conclusion

In this subanalysis of the EMIT-AF/VTE programme, patients’ edoxaban regimens were interrupted more frequently and for longer periods of time for major vs. nonmajor surgeries. Periprocedural management of edoxaban driven by decisions from the attending clinicians was associated with low rates of all bleeding, major bleeding, and CRNMB and thromboembolic events in patients undergoing major or nonmajor surgeries.

## Data Availability

The data underlying this article cannot be shared publicly, as the Global EMIT-AF/VTE study is currently ongoing.

## Abbreviations

ACS: Acute coronary syndrome
AF: Atrial fibrillation
CHA_2_DS_2_-VASc: Congestive heart failure, Hypertension, Age ≥75 (doubled), Diabetes, Stroke (doubled), Vascular disease, Age 65–74 years, and Sex category (female)
CrCL: Creatinine clearance
CRNMB: Clinically relevant nonmajor bleeding
CV: Cardiovascular
DOACs: Direct oral anticoagulants
EHRA: European Heart Rhythm Association
HAS-BLED: Hypertension, Abnormal renal/liver function, Stroke, Bleeding history or predisposition, Labile international normalised ratio, Elderly, Drugs/alcohol concomitantly
ISTH: International Society of Thrombosis and Haemostasis
SEEs: Systemic embolic events
SD: Standard deviation
VTE: Venous thromboembolism
